# SPECT/CT imaging of lower extremity perfusion reserve: A non-invasive correlate to exercise tolerance and cardiovascular fitness in patients undergoing clinically indicated myocardial perfusion imaging

**DOI:** 10.1007/s12350-019-02019-w

**Published:** 2020-01-14

**Authors:** Ting-Heng Chou, Sarah Janse, Albert J. Sinusas, Mitchel R. Stacy

**Affiliations:** 1 Department of Internal Medicine, Yale University School of Medicine, New Haven, CT USA; 2 Center for Regenerative Medicine, The Research Institute at Nationwide Children’s Hospital, 575 Children’s Crossroad, WB4131, Columbus, OH 43215 USA; 3 Department of Biomedical Informatics, The Ohio State University College of Medicine, Columbus, OH USA; 4 Department of Radiology & Biomedical Imaging, Yale University School of Medicine, New Haven, CT USA; 5 Division of Vascular Diseases and Surgery, Department of Surgery, The Ohio State University College of Medicine, Columbus, OH USA

**Keywords:** SPECT, hybrid imaging, MPI, vascular imaging, exercise testing, perfusion agents

## Abstract

**Background:**

Although exercise is often prescribed for the management of cardiovascular diseases, a non-invasive imaging approach that quantifies skeletal muscle physiology and correlates with patients’ functional capacity and cardiovascular fitness has been absent. Therefore, we evaluated the potential of lower extremity single photon emission computed tomography (SPECT)/CT perfusion imaging as a non-invasive correlate to exercise tolerance and cardiovascular fitness.

**Methods:**

Patients (*n* = 31) undergoing SPECT/CT myocardial perfusion imaging underwent additional stress/rest SPECT/CT imaging of the lower extremities. CT-based image segmentation was used for regional quantification of perfusion reserve within the tibialis anterior, soleus, and gastrocnemius muscles. Metabolic equivalents (METs) at peak exercise and heart rate recovery (HRR) after exercise were recorded.

**Results:**

Peak METs were significantly associated with perfusion reserve of tibialis anterior (*p* = 0.02), soleus (*p* = 0.01) and gastrocnemius (*p* = 0.01). HRR was significantly associated with perfusion reserve of the soleus (*p* = 0.02) and gastrocnemius (*p* = 0.04) muscles. Perfusion reserve of the tibialis anterior (40.6 ± 20.2%), soleus (35.4 ± 16.7%), and gastrocnemius (29.7 ± 19.1%) all significantly differed from each other.

**Conclusions:**

SPECT/CT imaging provides regional quantification of skeletal muscle perfusion reserve which is significantly associated with exercise tolerance and cardiovascular fitness. Future application of SPECT/CT may elucidate the underlying skeletal muscle adapations to exercise therapy in patients with cardiovascular diseases.

**Electronic supplementary material:**

The online version of this article (10.1007/s12350-019-02019-w) contains supplementary material, which is available to authorized users.

## Spanish Abstract

**Antecedentes:**

la actividad física a menudo se prescribe como parte del tratamiento de enfermedades cardiovasculares, sin embargo, en la actualidad no existe un enfoque de evaluación no invasiva que cuantifique la fisiología del músculo esquelético y se correlacione con la capacidad funcional y el estado cardiovascular de los pacientes. Nosotros evaluamos el potencial de la evaluación no invasiva de la extremidad inferior con técnica de tomografía computarizada por emisión de fotón único (SPECT/CT), y su correlación con la tolerancia al ejercicio y el estado cardiovascular.

**Métodos:**

todos los pacientes (*n* = 31) sometidos a adquisición de imágenes de perfusión miocárdica con técnica SPECT/CT fueron sometidos a adquisición de imágenes SPECT/CT estrés-reposo adicionales de las extremidades inferiores. Se utilizó la segmentación de imágenes basada en CT para la cuantificación regional de la reserva de perfusión dentro de los músculos tibial anterior, sóleo y gastrocnemio. Se registraron los equivalentes metabólicos (MET) en el ejercicio máximo y la recuperación de la frecuencia cardíaca (HRR) después del ejercicio.

**Resultados:**

los MET máximos se asociaron significativamente con la reserva de perfusión del músculo tibial anterior (*p* = 0.02) y el sóleo (*p* = 0.013) y el gastrocnemio (*p* = 0.01). La HRR se asoció significativamente con la reserva de perfusión de los músculos tibial anterior (*p* = 0.04), sóleo (*p* = 0.002) y gastrocnemio (*p* = 0.041). La reserva de perfusión del músculo tibial anterior (40,6 ± 20,2%), sóleo (35,4 ± 16,7%) y gastrocnemio (29,7 ± 19,1%) diferían significativamente entre sí.

**Conclusiones:**

la adquisición de imágenes por técnica SPECT/CT proporcionan una cuantificación regional de la reserva de perfusión del músculo esquelético, que se asocia significativamente con la tolerancia al ejercicio y el estado cardiovascular. El uso de éste método puede, en un futuro, dilucidar las adaptaciones subyacentes del músculo esquelético a la terapia física de pacientes con enfermedades cardiovasculares.

## Chinese Abstract

**背景:**

尽管运动处方是心血管疾病的一种常用治疗手段,但目前还缺乏可以定量评估骨骼肌生理功能,并反映患者体能和心肺适能的无创性影像方法。因此,我们采用下肢单光子发射断层扫描(SPECT)/ CT灌注成像的无创性方法,研究其对评估运动耐力和心肺适能的潜在应用价值。

**方法:**

选择31例行SPECT / CT心肌灌注显像的患者,进行额外的下肢负荷/静息SPECT / CT显像。 定量测定基于CT图像分割的胫前肌,比目鱼肌和腓肠肌的灌注储备。 记录运动高峰时的代谢当量(METs)和运动后的心率恢复(HRR)。

**结果:**

METs与胫前肌(*p* = 0.02),比目鱼肌(*p* = 0.01)和腓肠肌(*p* = 0.01)的灌注储备均显著相关。 HRR与比目鱼肌(*p* = 0.02)和腓肠肌(*p* = 0.04)的灌注储备均显著相关。 胫前肌(40.6 ± 20.2%),比目鱼肌(35.4 ± 16.7%)和腓肠肌(29.7 ± 19.1%)的灌注储备之间均存在显著差异。

**结论:**

SPECT / CT显像可定量分析骨骼肌的灌注储备,这与运动耐量和心肺适能密切相关。SPECT / CT可能对阐明心血管疾病患者运动治疗的骨骼肌适应作用具有潜在应用价值。

## French Abstract

**Contexte:**

Bien que l’exercice physique soit souvent prescrit dans le traitement des maladies cardiovasculaires, une approche d’imagerie non invasive qui quantifie la physiologie des muscles squelettiques et qui corrèle avec la capacité fonctionnelle des patients et leur capacité cardiovasculaire n’est pas disponible. Dans cette étude, nous avons évalué le potentiel de l’imagerie par perfusion tomodensitométrie à émission de photons (SPECT/CT) du membre inférieur comme corrélat non invasif à la tolérance à l’exercice et à la capacité cardiovasculaire.

**Méthodes:**

Les patients (*n* = 31) bénéficiant d’une imagerie de perfusion myocardique SPECT/CT ont également été étudiés au niveau des membres inférieures au repos et à l’effort par imagerie SPECT/CT. Nous avons utilisé la segmentation des images tomodensitométriques pour évaluer quantitativement la réserve régionale de perfusion des muscles tibial antérieur, soléaire et gastrocnémien. Les équivalents métaboliques (EMET) au pic d’exercice et lors de la récupération de la fréquence cardiaque (RFC) après l’exercice ont été enregistrés.

**Résultats:**

Les EMET maximaux furent significativement associés à la réserve de perfusion du tibial antérieur (*p* = 0,02), du soléaire (*p* = 0,013) et du gastrocnémien (*p* = 0,01). La RFC corréla avec la réserve de perfusion des muscles tibial antérieur (*p* = 0,04), soléaire (*p* = 0,002) et gastrocnémien (*p* = 0,041). Les valeurs de réserve de perfusion des muscles tibial antérieur (40,6 ± 20,2%), soléaire (35,4 ± 16,7%) et gastrocnémien (29,7 ± 19,1%) se sont avérées différentes.

**Conclusions:**

l’imagerie SPECT/CT permet la quantification de la réserve de perfusion régionale du muscle squelettique. Celle-ci est statistiquement et positivement associée à la tolérance à l’exercice et à la capacité cardiovasculaire. L’application future de SPECT/CT pourrait élucider les adaptations sous-jacentes des muscles squelettiques à la thérapie par l’exercice chez les patients atteints de maladies cardiovasculaires.

## Introduction

Exercise is often prescribed for clinical management of cardiovascular diseases with limited understanding of the physiological processes responsible for exercise-induced improvements in functional capacity, which is partially due to many clinical tests being unable to non-invasively evaluate physiological changes that occur within skeletal muscle in response to exercise therapy. For example, exercise training improves walking capacity in patients with peripheral artery disease (PAD);^1^ however, standard clinical physiological indices that reflect large vessel disease, such as the ankle-brachial index (ABI), have proven to be inconsistently correlated with measures of lower extremity function.^2^–^8^ Discrepancy between hemodynamic gradients in the extremities and functional measures may be explained by the fact that the mechanisms underlying exercise-induced functional improvements are unrelated to early changes in macrovasculature hemodynamics and instead reflect changes at the microvascular level. Potential mechanisms by which functional improvements occur include decreased levels of inflammation,^9^ increased endothelium-dependent vasodilation^10^ and capillary density,^11^ and/or an alteration in skeletal muscle metabolism,^12^ with many of these mechanisms being directly or indirectly associated with skeletal muscle perfusion. Therefore, a non-invasive approach that is capable of quantifying skeletal muscle perfusion and represents a composite index of changes in both the macrovasculature and microvasculature may correlate better with functional capacity and could assist in elucidating the underlying exercise-induced mechanisms of functional improvement, thereby improving the evaluation of exercise therapy for various forms of cardiovascular disease, such as PAD.

Previous studies that investigated the relationship between non-invasive perfusion assessment tools and lower extremity functional capacity have produced inconsistent results.^5^,^13^ Specifically, Szuba et al^5^ utilized plethysmography and found a non-significant relationship between baseline or hyperemic calf perfusion and walking distance, while Anderson et al^13^ showed first-pass contrast-enhanced magnetic resonance imaging (MRI) of calf perfusion during peak plantar flexion exercise was significantly correlated with 6-minute walking distance. One major limitation of these previously applied non-invasive techniques has been their inability to quantify skeletal muscle perfusion during normal walking or exercise stress conditions. In contrast, radiotracer-based imaging, which allows for non-invasive assessment of the composite changes in macrovascular and microvascular perfusion under dynamic exercise (i.e., walking and running) conditions, may be an ideal non-invasive imaging tool for evaluating the relationship between skeletal muscle perfusion and functional capacity, thereby providing novel insight into physiological adaptations to exercise interventions. Previous radiotracer-based imaging studies have utilized two-dimensional (2D) scintigraphy with thallium-201^14^–^17^ and technetium-99m (^99m^Tc)-sestamibi^18^–^21^ to assess lower extremity skeletal muscle perfusion under conditions of rest and exercise stress and demonstrated the utility of this approach for detecting abnormalities in muscle perfusion; however, to date, these radiotracer-based approaches have not been utilized to evaluate the relationship between regional three-dimensional (3D) calf muscle perfusion and functional capacity.

The purpose of the present study was to evaluate the utility of lower extremity SPECT/CT perfusion imaging as a non-invasive correlate to lower extremity functional capacity and cardiovascular fitness. To accomplish this goal, we incorporated additional SPECT/CT perfusion imaging of the calves in patient's already prescribed routine Bruce protocol treadmill stress tests for the assessment of stress/rest myocardial perfusion, which eliminated the need for an additional injection of radiotracer and repeat exercise stress testing. Peak metabolic equivalents (METs) and heart rate recovery (HRR) were used as indicators of exercise tolerance and cardiovascular fitness, as both are common measures acquired during exercise stress testing and also serve as indicators of long-term mortality.^22^,^23^ We hypothesized that lower extremity SPECT/CT perfusion imaging would allow for regional assessment of skeletal muscle perfusion reserve and would be significantly associated with both exercise capacity and cardiovascular fitness.

## Methods

### Research Subjects

Patients (*n* = 31) who were scheduled to undergo clinically indicated exercise stress and rest myocardial perfusion SPECT/CT imaging due to prior chest pain, angina, dyspnea, and/or abnormal ECG were recruited from the nuclear cardiology clinical laboratory at Yale New Haven Hospital. The study protocol was approved by the Institutional Review Board for Human Subjects Research and Review Committee as well as the Radiation Safety Committee and was in accordance with the guidelines set forth by the Declaration of Helsinki. All individuals provided written informed consent after receiving an explanation of the experimental procedures and potential risks associated with participating in the study.

### Exercise Stress Test Protocol

Patients arrived at the nuclear cardiology laboratory for clinically indicated stress/rest myocardial perfusion imaging following a 4-hour fast and abstinence from caffeine and alcohol for at least 12 hours. Patients had a catheter placed in an antecubital vein for radiopharmaceutical administration and were prepped for rest 12-lead ECG and stress ECG monitoring. Following baseline assessment of resting heart rate, blood pressure, and ECG, a standard Bruce protocol treadmill exercise stress test was performed. One to two minutes prior to the termination of maximum peak exercise, a low dose (326.3 ± 53.3 MBq) of ^99m^Tc-tetrofosmin (Myoview; GE Healthcare) was intravenously administered for peripheral and myocardial stress SPECT/CT perfusion imaging. The endpoint of the exercise test was determined based on when patients demonstrated symptoms such as fatigue, moderate to severe chest pain, excessive shortness of breath, dramatic ST depressions (> 2 mm) or ST elevations (> 1 mm), or achievement of adequate stress by heart rate criteria. The heart rate criteria for a successful stress test was defined as the patient achieving 85% (75% for patients prescribed a beta blocker) of age-predicted maximum heart rate (i.e., 220-age). After termination of the stress test, patients immediately began slowly walking on the treadmill in a cooldown phase for approximately 1-2 minutes at a speed of 2.5 km per hour and zero degree slope and then rested in the supine position for approximately 15-45 minutes before cardiac imaging, as per American Society of Nuclear Cardiology (ASNC) guidelines. The peak workload achieved, defined as the peak METs attained during peak exercise, was recorded for each patient. HRR, calculated as the heart rate at peak exercise minus the heart rate 1 minute after the cessation of exercise, was also recorded for 27 of the 31 patients.

### Cardiac SPECT/CT Imaging Protocol

Stress and rest SPECT/CT myocardial perfusion images (MPI) were acquired in list mode using a dedicated cardiac SPECT/CT camera that was composed of 19 solid-state cadmium zinc telluride gamma detectors with 5 mm pinhole collimators as well as a 64-slice CT system for SPECT attenuation correction (Discovery NM/CT 570c, GE Healthcare). Stress/rest cardiac gated SPECT images were acquired with each patient’s heart centered in the field-of-view of the SPECT detectors using a 140.5 keV ± 10% window for ^99m^Tc-tetrofosmin. Immediately following the SPECT acquisition, CT images were acquired with a slice thickness of 2.5 mm, at 120 kVp, and 50 mA for the purposes of SPECT attenuation correction. SPECT images were reconstructed with commercially available software (Xeleris, GE Healthcare, Buckinghamshire, UK) using a matrix size of 70x70 pixels (pixel size, 4 mm). Reconstructed SPECT images were processed using Yale SPECT image quantification software for the evaluation of myocardial perfusion defect sizes based on a 17-segment model.^24^ Patients were grouped as “normal MPI” or “abnormal MPI” based on previously published methodology where the combination of a board certified nuclear cardiologist’s image interpretation and a quantified summed stress score (SSS) ≥ 3 were used as criteria for defining abnormal MPI.^25^,^26^

### Lower Extremity SPECT/CT Imaging Protocol

**Figure 1:**
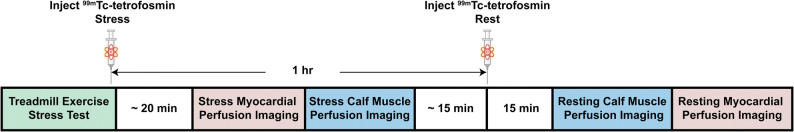
SPECT/CT imaging timeline and protocol for evaluating lower extremity skeletal muscle perfusion reserve in patients undergoing clinically indicated myocardial perfusion imaging. As shown by the figure, the evaluation of lower extremity perfusion did not increase the length of time spent in the clinic and did not require any additional radiotracer injections

Following exercise stress testing and clinically indicated stress myocardial perfusion imaging, patients were immediately transferred to a conventional hybrid SPECT/4-slice CT imaging system with large field-of-view sodium iodide detectors (Infinia Hawkeye, GE Healthcare) for post stress SPECT/CT imaging of the lower extremities utilizing the previously administered stress dose of ^99m^Tc-tetrofosmin. After completion of stress imaging of the lower extremities, patients rested comfortably in the supine position until at least 1 hour of time had elapsed since administration of the initial stress low dose of ^99m^Tc-tetrofosmin. The second, higher dose of ^99m^Tc-tetrofosmin (693.4 ± 56.2 MBq) was then intravenously administered while the patient rested comfortably in the supine position, with subsequent resting SPECT/CT imaging of the lower extremities performed 15 minutes after radiotracer administration. Patients then underwent resting cardiac SPECT/CT imaging as part of their clinically indicated exam within the 30-60 minutes after radiotracer injection as per ASNC guidelines. A detailed summary of the SPECT/CT imaging workflow is provided in Figure 1.

**Figure 2:**
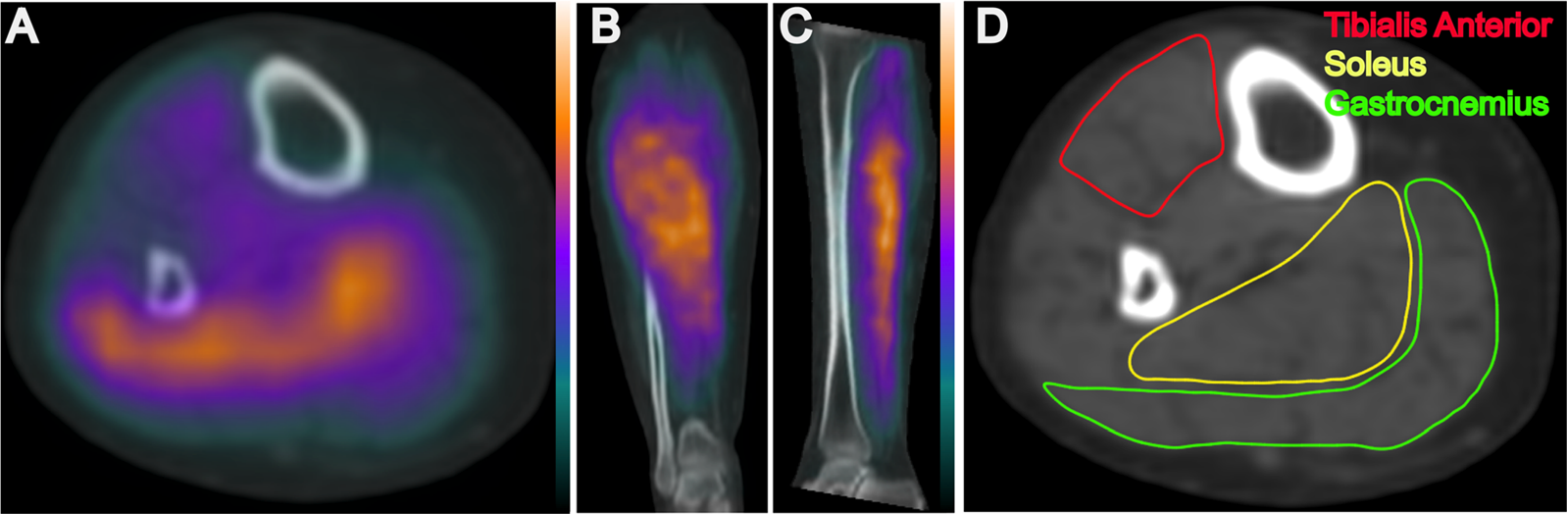
SPECT/CT imaging of lower extremity perfusion and CT-based image segmentation for regional perfusion analysis. Representative **A** transaxial, **B** coronal, and **C** sagittal views of SPECT/CT perfusion imaging of the calf. **D** Image segmentation of specific calf muscles of interest from a low-dose CT attenuation scan

All SPECT images of the lower extremities were acquired using a 360º step and shoot acquisition with a 140.5 keV ± 10% window, 3º projections, and 30 s per stop. Immediately following the SPECT acquisition, CT images were acquired with a slice thickness of 5 mm at 140 kVp and 2.5 mA for the purposes of SPECT attenuation correction and future image segmentation of calf muscles. All SPECT images were reconstructed using 2 iterations and 10 subsets of the ordered subset expectation and maximization (OSEM) algorithm, applying corrections for attenuation, scatter, and resolution loss. SPECT/CT images were reconstructed using system software (Xeleris, GE Healthcare, Buckinghamshire, UK) that facilitated the generation of co-registered SPECT and CT images of the calves (Figure 2A–C).

### Lower Extremity SPECT/CT Image Processing

SPECT/CT images were analyzed using a commercially available image analysis toolkit (OsiriX, Pixmeo SARL, Bernex, Switzerland) that allowed for image segmentation and regional assessment of calf muscle perfusion. Low-dose CT attenuation images were used to define and manually segment individual calf muscles of interest (tibialis anterior, soleus, and gastrocnemius) from both the left and right lower extremity of each patient (Figure 2D). Due to patients remaining on the scanner table between stress and rest imaging of the lower extremities, no change in patient or bed position occurred between scans, which allowed for the same volumes of interest (VOIs) to be utilized for both stress and rest SPECT/CT image analysis. Average ^99m^Tc-tetrofosmin uptake was quantified from co-registered SPECT images within the CT-defined VOIs. Average SPECT image intensity values were normalized to injected radiotracer dose (mCi) and patient body weight (kg). Perfusion reserve of the calf muscles was calculated as the relative percent change in ^99m^Tc-tetrofosmin uptake from resting to stress condition.

### Statistical Analysis

Continuous variables were calculated as means and standard deviations for normally distributed variables and as medians and interquartile range for non-normally distributed variables. Categorical variables were calculated as counts and percentages. Associations with variables of interest and the three outcomes of perfusion reserve for the tibialis anterior, soleus, and gastrocnemius were tested using linear mixed models with a random effect for patient. From these models, estimates with 95% confidence intervals and *p*-values were calculated for each predictor and multivariate analyses that were performed to adjust for age and gender when estimating associations between lower extremity perfusion reserve and both HRR and METs. Estimates for continuous variables are for a change in value of 1 standard deviation. A linear mixed model with a random effect for subject was also used to test for differences in perfusion reserve between muscles, followed by pairwise comparisons to test for differences in perfusion reserve between each pair of muscles. To account for multiple comparisons, Tukey’s method was used to preserve an overall type I error rate of 0.05. A *p* value of < 0.05 was considered statistically significant. All statistical analyses were performed using SAS, version 9.4 (SAS Institute Inc., Cary, NC).

## Results

### Patient Characteristics

The patient population (mean age: 61 ± 8 years) was composed of 81% males and 19% females. Among these patients, 42% had a history of coronary artery disease, 74% were hypertensive, 68% had hyperlipidemia, 13% had type 2 diabetes mellitus (DM), and 16% were tobacco users at the time of study enrollment. Additionally, three patients had a history of prior myocardial infarction or percutaneous coronary intervention. No patient had a history of claudication or PAD, and none had prior ABI measurements. Among those patients on medications, 12 of 31 patients were taking beta blockers at the time of study enrollment, 2 patients were taking both beta and calcium blockers, and 1 patient was prescribed a calcium blocker alone. A complete summary of patient demographics and the clinical indications for stress testing are provided in Table 1.

**Table 1:** Subject demographics

Patient characteristics	
Age (years)	61 ± 8
Male/female	25/6
Body mass index (kg/m^2^)	29.3 ± 4.4
Peak METs	10.3 ± 2.5
Resting HR (bpm)	69 ± 14
Peak stress HR (bpm)	148 ± 17
Abnormal MPI	10 (32%)
Coronary artery disease	13 (42%)
Hypertension	23 (74%)
Hyperlipidemia	21 (68%)
Diabetes mellitus	4 (13%)
Tobacco use	5 (16%)
Clinical indication for stress testing	
Chest pain	16 (52%)
Dyspnea	5 (16%)
Abnormal ECG	4 (13%)
Coronary atherosclerosis	3 (10%)
Post MI or PCI	3 (10%)

*N* = 31 patients. Values are mean ± SD or number of patients (percentage)

*METs*, metabolic equivalents; *HR*, heart rate; *MPI*, myocardial perfusion imaging; *MI*, myocardial infarction; *PCI*, percutaneous coronary intervention

### Treadmill Exercise Stress Test

The average total treadmill exercise time during the Bruce protocol was 8.9 ± 2.1 minutes, which translated to an average peak METs of 10.3 ± 2.5 being attained during exercise stress testing. The mean systolic and diastolic blood pressure (SBP/DBP) increased from 138.1 ± 16.6/82.0 ± 10.1 mmHg at rest to 183.2 ± 18.6/88.6 ± 10.8 mmHg during peak exercise. On average, patient's heart rate increased from 69 ± 14 bpm at rest to 148 ± 17 bpm during peak exercise. Following cessation of peak exercise, mean HRR was 38 ± 14 bpm, which was found to be significantly correlated with peak METs attained during treadmill exercise (*r *= 0.42, *p* = 0.03).

### SPECT/CT Imaging of Myocardial Perfusion

Based on image interpretation and quantitative analysis of cardiac SPECT/CT imaging, 10 out of 31 patients were found to have abnormal MPI. Among the 10 patients with abnormal MPI results, 4 patients had ischemic ECG changes and no patients had transient ischemic dilation of the left ventricle. No significant diffference existed between patients with normal and abnormal MPI with regard to peak METs attained during exercise stress testing (normal MPI: 10.5 ± 2.5 METs, abnormal MPI: 10.0 ± 2.4 METs; *p* = 0.6). Additionally, no differences in lower extremity perfusion reserve existed for any calf muscle groups when comparing normal and abnormal MPI patient groups (tibialis anterior: *p* = 0.99; soleus: *p* = 0.98; gastrocnemius: *p* = 0.99). Futher analysis of the potential adverse effect of abnormal MPI on lower extremity perfusion reserve demonstrated that MPI result was not significantly associated with the lower extremity perfusion reserve of any calf muscle group (tibialis anterior, *p* = 0.6; soleus, *p* = 0.3; gastrocnemius, *p* = 0.5).

### SPECT/CT Imaging of Lower Extremity Perfusion Reserve

**Figure 3:**
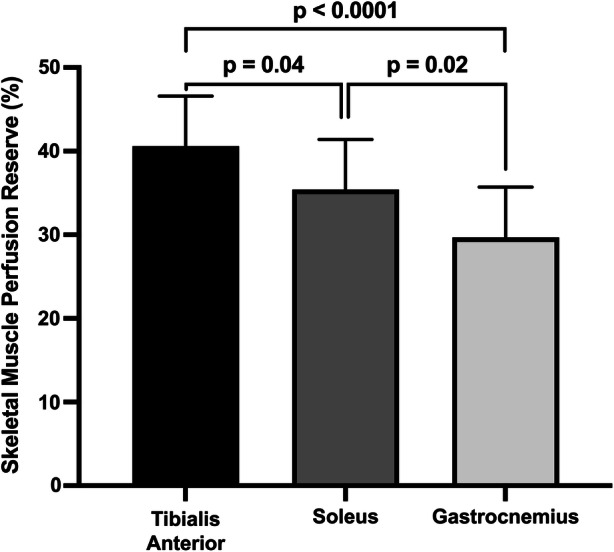
Regional evaluation of SPECT/CT-derived measures of lower extremity perfusion reserve. Perfusion reserve of the tibialis anterior, soleus, and gastrocnemius muscle groups significantly differed from each other. *N* = 31 patients

**Figure 4:**
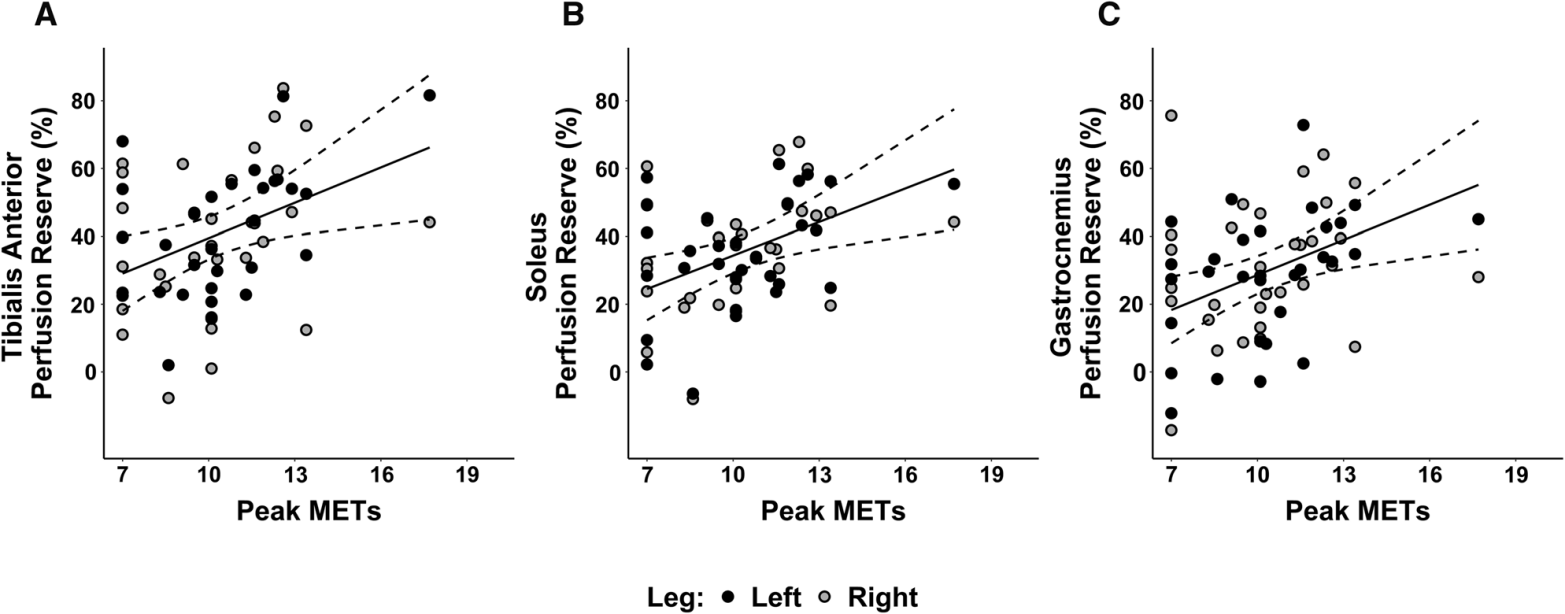
Relationship between skeletal muscle perfusion reserve and functional exercise capacity. Peak METs attained during treadmill stress test were significantly associated with SPECT/CT-derived measures of perfusion reserve in the **A** tibialis anterior (*p* = 0.02; marginal *R*^2^ $\left( {R_{{({\text{m}})}}^{ 2} } \right)$ = 0.20; conditional *R*^2^ $\left( {R_{{({\text{c}})}}^{ 2} } \right)$ = 0.72), **B** soleus (*p* = 0.01; $R_{{({\text{m}})}}^{ 2}$ = 0.26; $R_{{({\text{c}})}}^{ 2}$ = 0.90), and the **C** gastrocnemius muscle (*p* = 0.01; $R_{{({\text{m}})}}^{ 2}$ = 0.24 $R_{{({\text{c}})}}^{ 2}$ = 0.67). Solid lines represent predicted values of perfusion reserve and dotted lines represent 95% confidence intervals for the predicted fit. Points represent the quantified data from 31 patients

**Figure 5:**
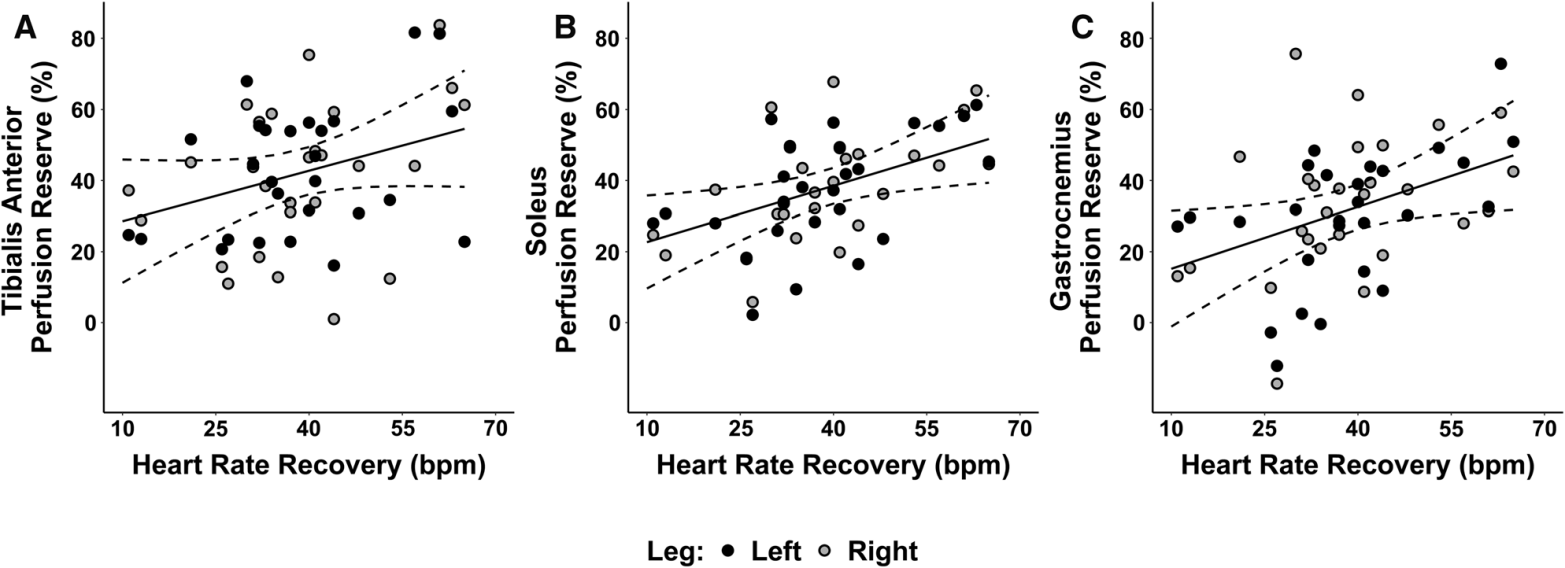
Relationship between skeletal muscle perfusion reserve and cardiovascular fitness. Heart rate recovery after treadmill stress testing was not significantly associated with SPECT/CT-derived measures of perfusion reserve in the **A** tibialis anterior (*p* = 0.1; $R_{{({\text{m}})}}^{ 2}$ = 0.13; $R_{{({\text{c}})}}^{ 2}$ = 0.69), but was significantly associated with perfusion reserve of the **B** soleus (*p* = 0.02; $R_{{({\text{m}})}}^{ 2}$ = 0.29; $R_{{({\text{c}})}}^{ 2}$ = 0.89) and gastrocnemius (*p* = 0.04; $R_{{({\text{m}})}}^{ 2}$ = 0.19; $R_{{({\text{c}})}}^{ 2}$ = 0.68) muscle groups. Solid lines represent predicted values of perfusion reserve and dotted lines represent 95% confidence intervals for the predicted fit. Points represent the quantified data from 27 patients

SPECT/CT imaging-derived measures of calf muscle perfusion reserve for the tibialis anterior, soleus, and gastrocnemius muscle groups were 40.6 ± 20.2%, 35.4 ± 16.7%, and 29.7 ± 19.1%, respectively. Addtionally, each calf muscle group significantly differed from each of the other muscle groups. Perfusion reserve of the tibialis anterior was on average 10.9% higher than gastrocnemius (*p* < 0.0001) and 5.2% higher than the soleus (*p* = 0.04), and perfusion reserve of the soleus was on average 5.7% higher than the gastrocnemius (*p* = 0.02) (Figure 3). An increase in peak METs was significantly associated with an increase in perfusion reserve of the tibialis anterior (*p* = 0.02), soleus (*p* = 0.01), and gastrocnemius (*p* = 0.01). Specifically, an increase in one standard deviation of peak METs (i.e., 2.4) was associated with an average increase in perfusion reserve of 8.5% (standard error (SE) = 3.4%; *p* = 0.02), 8.0% (SE = 2.8%; *p* = 0.01), and 8.4% (SE = 3.0%; *p* = 0.01) in the tibialis anterior, soleus, and gastrocnemius muscles, respectively (Figure 4). In addition to markers of exercise tolerance significantly associating with calf muscle perfusion reserve, HRR was also found to be significantly associated with calf muscle perfusion reserve. Specifically, an increase in one standard deviation of HRR (i.e., 13.5 beats/minute) was associated with an average increase in perfusion reserve of 7.1% (SE = 2.9%; *p* = 0.02) and 7.8% (SE = 3.6%; *p* = 0.04) in the soleus and gastrocnemius muscles, respectively, but was not significantly associated with the tibialis anterior (SE = 3.8%; *p* = 0.1) (Figure 5). Age was not statistically significant in any of the adjusted models, while gender was only significant (*p* = 0.02) in the model evaluating the association between HRR and perfusion reserve of the gastrocnemius.

Analysis of the potential association between clinical indication for stress testing and lower extremity perfusion reserve revealed that chest pain, abnormal ECG, coronary atherosclerosis, and prior MI or PCI were not significantly associated with perfusion reserve of any muscle group (*p* > 0.05), while dyspnea was the only clinical indication that was found to be significantly associated with perfusion reserve of any muscle group of interest (gastrocnemius, *p* = 0.04). Evaluation of the potential effect of type 2 DM on perfusion reserve revealed that patients with DM (*n* = 4) did not significantly differ from patients without DM (*n* = 27) with regard to perfusion reserve of the gastrocnemius (*p* = 0.3), soleus (*p* = 0.9), and tibialis anterior (*p* = 0.4).

Additional analysis of perfusion reserve between right and left legs demonstrated that perfusion reserve values for each muscle group of the right and left leg were significantly correlated with each other (gastrocnemius: *r *= 0.68, *p* < 0.0001; soleus: *r *= 0.89, *p* < 0.0001; tibialis anterior: *r *= 0.71, *p* < 0.0001). Right and left leg comparisons showed that no significant differences in perfusion reserve existed between legs for any of the muscle groups of interest (gastrocnemius: *p* = 0.4; soleus: *p* = 0.4; tibialis anterior: *p* = 0.7).

## Discussion

While exercise therapy is utilized for the management of various forms of cardiovascular disease, quantitative imaging tools to serially assess exercise-induced alterations in skeletal muscle physiology have been lacking. A non-invasive approach that quantifies regional physiological responses to exercise stress and correlates with functional capacity could assist clinicians in understanding the underlying adaptations that occur in skeletal muscle in response to exercise therapy in patients with cardiovascular disease. In the current study, we have demonstrated that quantitative evaluation of skeletal muscle perfusion reserve can be achieved by incorporating additional lower extremity SPECT/CT imaging into routine one-day stress/rest SPECT/CT myocardial perfusion imaging protocols without the need for additional radiotracer injection or exercise stress testing. Additionally, we found that SPECT/CT-derived measures of perfusion reserve are muscle group dependent and for the first time demonstrated that calf muscle perfusion reserve is significantly associated with markers of functional exercise tolerance (peak METs) and cardiovascular fitness (HRR).

Although other non-invasive imaging tools such as MRI and positron emission tomography (PET) provide absolute quantification of muscle perfusion, these modalities to date have only been used for the assessment of skeletal muscle perfusion during exercise paradigms that utilize and activate a limited quantity of muscle (i.e., knee extension, dorsiflexion, and plantar flexion exercise).^27^,^28^ Alternatively, SPECT perfusion radiotracers are commonly administered clinically during peak treadmill exercise for functional imaging of the heart and can also be utilized for complementary assessment of skeletal muscle perfusion in both lower extremities. In the present study, using a stress/rest SPECT/CT imaging protocol during standard treadmill stress testing, we demonstrate the ability of SPECT/CT imaging to quantify perfusion responses to functional treadmill walking/running exercise within individual muscle groups of the calf, which has not been previously achieved by MRI-, PET-, or SPECT-based imaging investigations. We found heterogeneous perfusion responses in the calf, with the tibialis anterior muscle group demonstrating the highest perfusion reserve (40.6 ± 20.2%), followed by the soleus (35.4 ± 16.7%) and gastrocnemius (29.7 ± 19.1%) muscles (Figure 3). These heterogeneous perfusion responses to treadmill exercise are in agreement with prior PET imaging studies that have also demonstrated heterogeneous perfusion in the calf in response to various forms of plantar/dorsiflexion exercise and showed exercise-induced increases in perfusion of the gastrocnemius and tibialis anterior muscle groups.^29^,^30^ The additional increase in perfusion reserve of the soleus muscle that was observed in the present study may be due to the type of functional activity that was performed (i.e., treadmill exercise), which likely required activation of additional muscle groups to achieve varying degrees of stability for different treadmill speeds and incline gradients. This ability to non-invasively characterize regional perfusion reserve in the lower extremities during functional treadmill exercise could possess utility for a variety of clinical conditions, such as screening for and identification of underlying perfusion abnormalities in patients with suspected PAD, as well as for assessing regional specific improvements in muscle perfusion in PAD patients following exercise interventions that are directed at improving claudication symptoms.

By demonstrating significant associations between exercise-induced changes in skeletal muscle perfusion within individual muscles of the calf and peak METs during treadmill exercise (Figure 4), the present study revealed that SPECT/CT perfusion imaging can serve as a non-invasive correlate to functional capacity. Thus, our data suggests that SPECT/CT perfusion imaging may possess potential utility for non-invasively evaluating the exercise-induced physiological adaptations that happen in skeletal muscle and correspond with concurrent functional improvements. In addition to significantly associating with functional capacity, the present study also revealed that SPECT/CT-derived measures of perfusion reserve are significantly associated with HRR after the completion of peak treadmill exercise (Figure 5). While the underlying mechanisms of this relationship are unclear, the interplay between muscle perfusion and HRR is likely related to the effect of parasympathetic activity on blood vessels of skeletal muscle or the overall cardiovascular fitness of patients. Indeed, the decrease in heart rate immediately after exercise is known to be a function of vagal reactivation.^31^ Postganglionic parasympathetic neurons release acetylcholine which binds to M3 muscarinic receptors located at the endothelial cells of blood vessels and eventually results in vasodilation.^32^ Therefore, vagal tone may affect both perfusion and HRR and thus contribute to the observed relationship in the present study. Alternatively, a specific amount of skeletal muscle perfusion may be required to achieve a specific workload (i.e., METs) during treadmill exercise. Therefore, patients who are more fit would presumably have higher perfusion reserve to achieve higher METs and possibly also have higher vagal tone and HRR.^33^ Given that HRR is a powerful predictor of overall mortality,^22^,^23^ the current results suggest that lower extremity skeletal muscle perfusion reserve may have important clinical relevance in patients with cardiovascular diseases, particularly in PAD patients who suffer from lower extremity perfusion deficits.

In addition to demonstrating significant associations between lower extremity perfusion reserve and functional exercise tolerance and cardiovascular fitness, the present study also found that perfusion reserve of the lower extremities was not significantly affected by the presence of abnormal myocardial perfusion and was not significantly associated with the results of MPI. These results are similar to the findings of a previous study by Scholtens et al,^34^ which found that PET-derived measures of perfusion reserve for the myocardium and peripheral skeletal muscle were not significantly correlated when using intravenous adenosine as a vasodilator stress agent. While the vasodilator effects of adenosine and treadmill exercise likely differ mechanistically, the current study provides further evidence that suggests a potential disconnect between the perfusion of the heart and the perfusion of the lower extremities.

Although the present study demonstrated the utility of SPECT/CT imaging for assessing lower extremity skeletal muscle perfusion reserve, the total patient enrollment was relatively small and patients did not have a history of PAD. Thus, application of SPECT/CT perfusion imaging in a wider spectrum of patients, such as patients with PAD and type 2 DM, may assist in understanding how underlying peripheral vascular conditions may lead to regional alterations in skeletal muscle perfusion reserve. Additionally, while we were able to detect peripheral calcification in a few patients, we were limited by poor spatial resolution (~ 5 mm) of the low-dose CT attenuation scans. Future investigations may benefit from using higher resolution CT imaging to elucidate the potential effect of peripheral calcification on lower extremity perfusion reserve.

## Conclusion

The present study demonstrates the utility of ^99m^Tc-tetrofosmin SPECT/CT imaging for quantifying regional changes in skeletal muscle perfusion in response to treadmill exercise in a clinically relevant population. Additionally, this study reveals the potential of radiotracer-based imaging for elucidating the underlying physiological adaptations that occur in response to exercise therapy. Future research is warranted to examine the relationships between SPECT/CT perfusion imaging, hemodynamic ABI measurements, cardiovascular fitness, and functional walking capacity in patients with cardiovascular disease to better understand the full clinical utility of SPECT/CT perfusion imaging for evaluating the benefits of therapeutic exercise interventions.

## New Knowledge Gained

Regional assessment of skeletal muscle perfusion reserve of the calf can be achieved in conjunction with clinically indicated myocardial perfusion imaging without the need for additional radiotracer injections, exercise stress testing, and time spent in the hospital. SPECT/CT-derived measures of lower extremity perfusion reserve significantly correlate with both exercise tolerance and cardiovascular fitness, thus making this imaging approach an attractive method for future studies focused on screening for PAD as well as for evaluating the physiological response to therapeutic exercise training programs.

## Electronic supplementary material

Below is the link to the electronic supplementary material.

Electronic supplementary material 1 (PPTX 2134 kb)

Electronic supplementary material 2 (M4A 1832 kb)

Electronic supplementary material 3 (DOCX 12 kb)

Electronic supplementary material 4 (TIFF 2992 kb)

## Abbreviations

PAD: Peripheral artery disease
ABI: Ankle-brachial index
SPECT: Single photon emission computed tomography
CT: Computed tomography
^99m^Tc: Technetium-99m
VOI: Volume of interest
METs: Metabolic equivalents
HRR: Heart rate recovery
