# Supplementary material for: SPECT/CT imaging of lower extremity perfusion reserve: A non-invasive correlate to exercise tolerance and cardiovascular fitness in patients undergoing clinically indicated myocardial perfusion imaging
Source: J Nucl Cardiol. 2020 Jan 14;27(6):1923–33. doi: 10.1007/s12350-019-02019-w (PMC7749094; doi:10.1007/s12350-019-02019-w)
Supplement: Supplementary file 1 — Electronic supplementary material 1 (PPTX 2134 kb) [file 12350_2019_2019_MOESM1_ESM.pptx]

## Slide 1
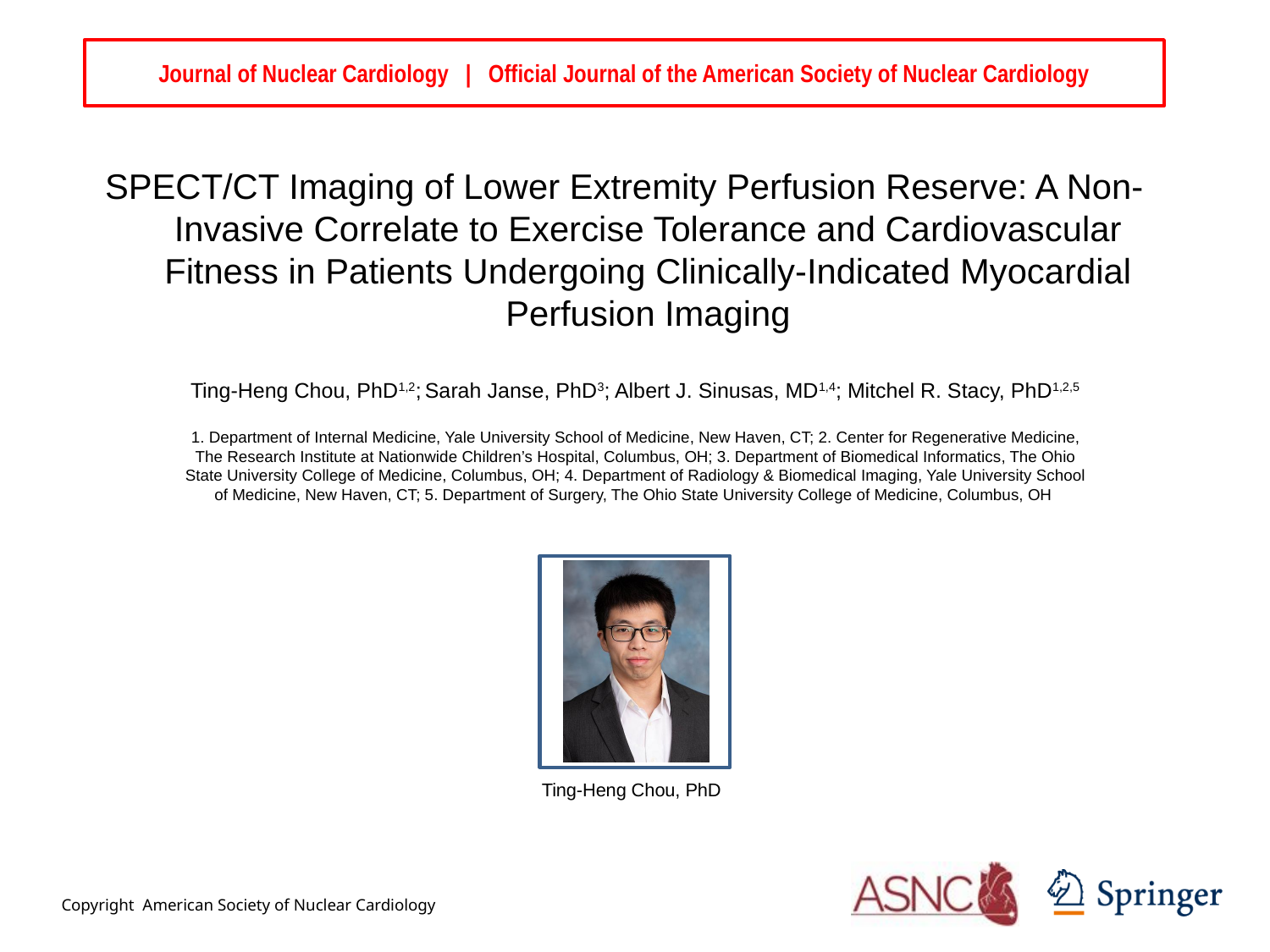

Journal of Nuclear Cardiology | Official Journal of the American Society of Nuclear Cardiology
# SPECT/CT Imaging of Lower Extremity Perfusion Reserve: A Non-Invasive Correlate to Exercise Tolerance and Cardiovascular Fitness in Patients Undergoing Clinically-Indicated Myocardial Perfusion Imaging
Ting-Heng Chou, PhD1,2; Sarah Janse, PhD3; Albert J. Sinusas, MD1,4; Mitchel R. Stacy, PhD1,2,5
1. Department of Internal Medicine, Yale University School of Medicine, New Haven, CT; 2. Center for Regenerative Medicine, The Research Institute at Nationwide Children’s Hospital, Columbus, OH; 3. Department of Biomedical Informatics, The Ohio State University College of Medicine, Columbus, OH; 4. Department of Radiology & Biomedical Imaging, Yale University School of Medicine, New Haven, CT; 5. Department of Surgery, The Ohio State University College of Medicine, Columbus, OH
Ting-Heng Chou, PhD
Copyright American Society of Nuclear Cardiology

## Slide 2
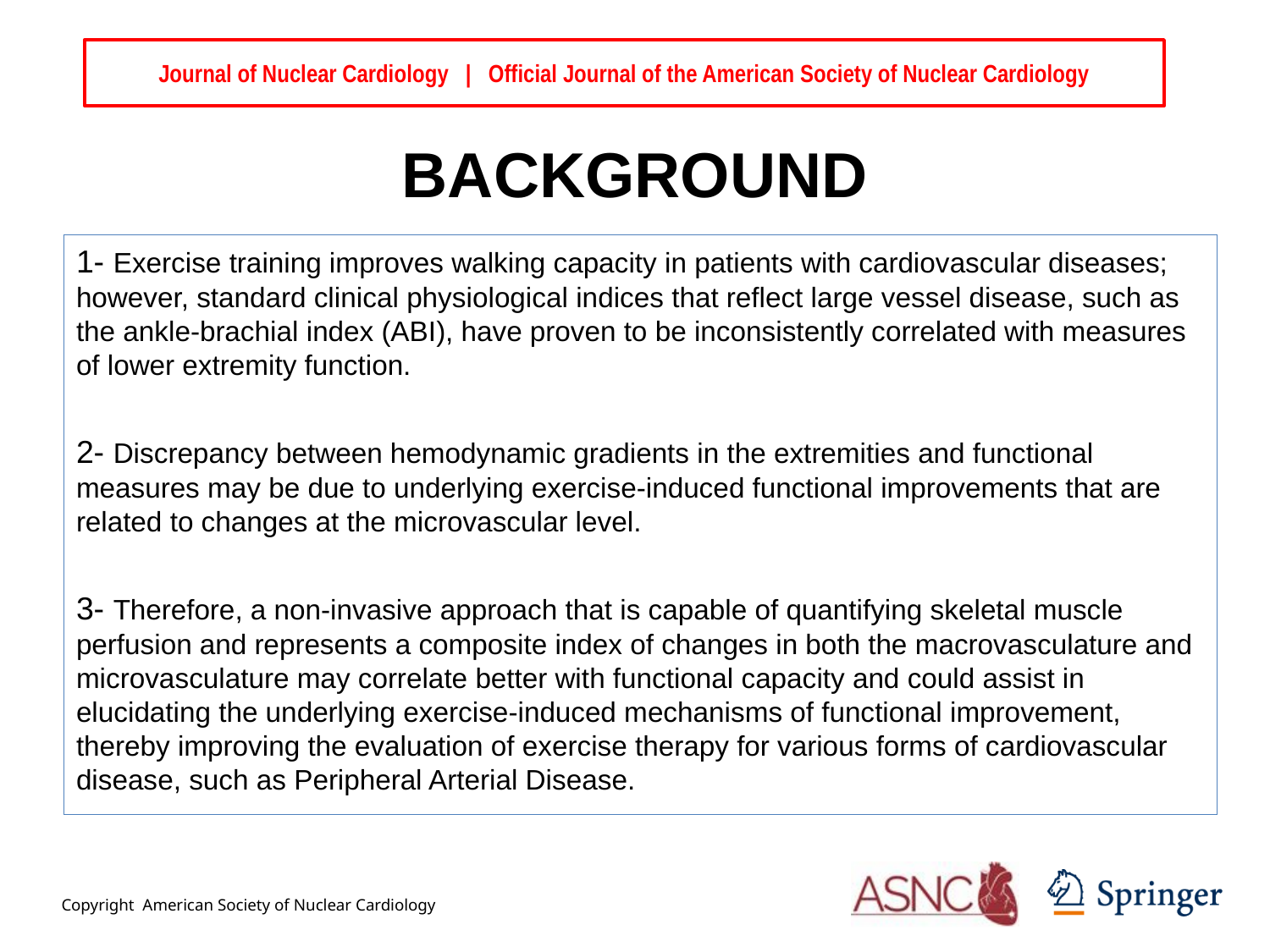

Journal of Nuclear Cardiology | Official Journal of the American Society of Nuclear Cardiology
# BACKGROUND
1- Exercise training improves walking capacity in patients with cardiovascular diseases; however, standard clinical physiological indices that reflect large vessel disease, such as the ankle-brachial index (ABI), have proven to be inconsistently correlated with measures of lower extremity function.
2- Discrepancy between hemodynamic gradients in the extremities and functional measures may be due to underlying exercise-induced functional improvements that are related to changes at the microvascular level.
3- Therefore, a non-invasive approach that is capable of quantifying skeletal muscle perfusion and represents a composite index of changes in both the macrovasculature and microvasculature may correlate better with functional capacity and could assist in elucidating the underlying exercise-induced mechanisms of functional improvement, thereby improving the evaluation of exercise therapy for various forms of cardiovascular disease, such as Peripheral Arterial Disease.
Copyright American Society of Nuclear Cardiology

## Slide 3
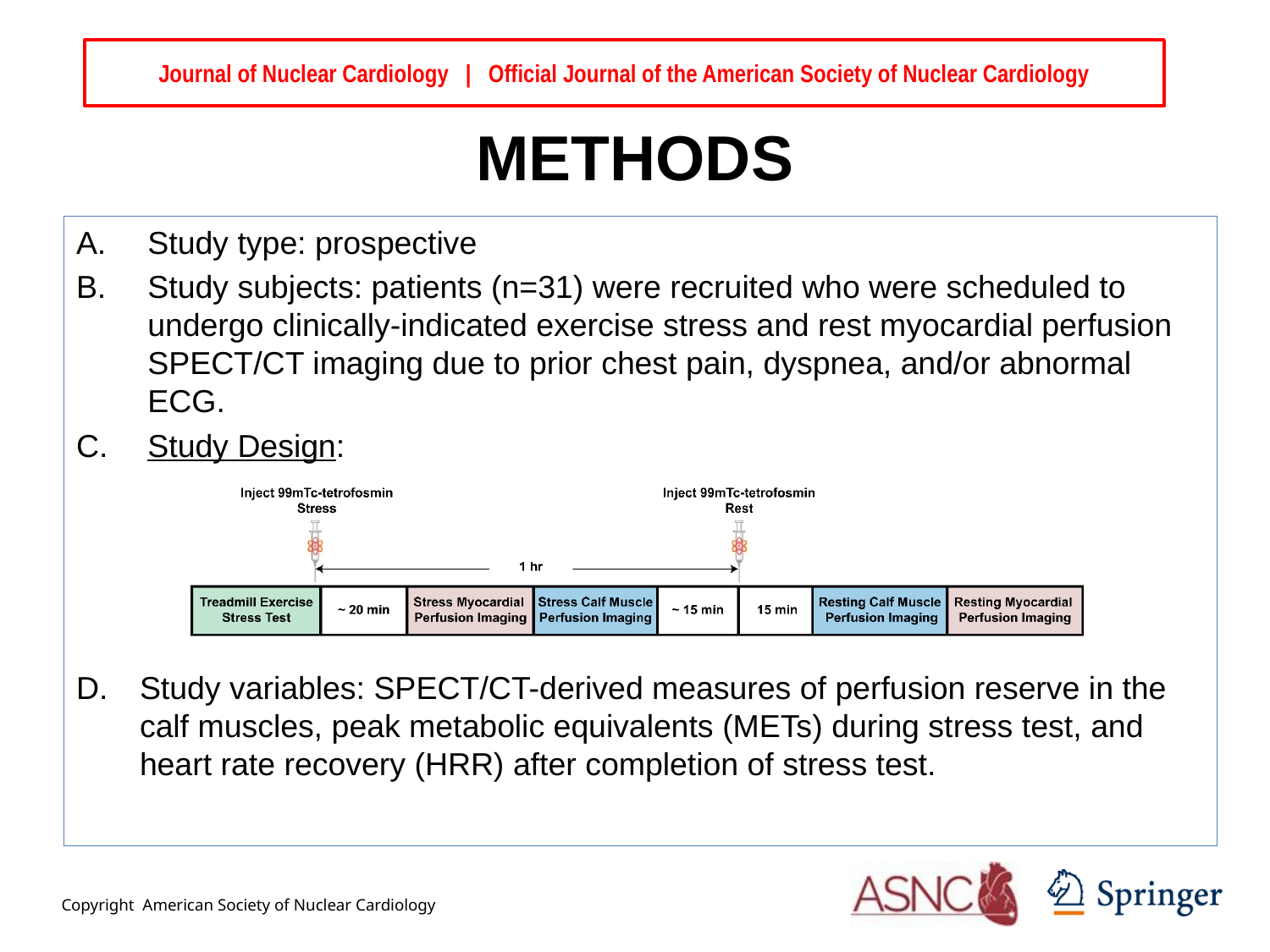

Journal of Nuclear Cardiology | Official Journal of the American Society of Nuclear Cardiology
# METHODS
Study type: prospective
Study subjects: patients (n=31) were recruited who were scheduled to undergo clinically-indicated exercise stress and rest myocardial perfusion SPECT/CT imaging due to prior chest pain, dyspnea, and/or abnormal ECG.
Study Design:
Study variables: SPECT/CT-derived measures of perfusion reserve in the calf muscles, peak metabolic equivalents (METs) during stress test, and heart rate recovery (HRR) after completion of stress test.
Copyright American Society of Nuclear Cardiology

## Slide 4
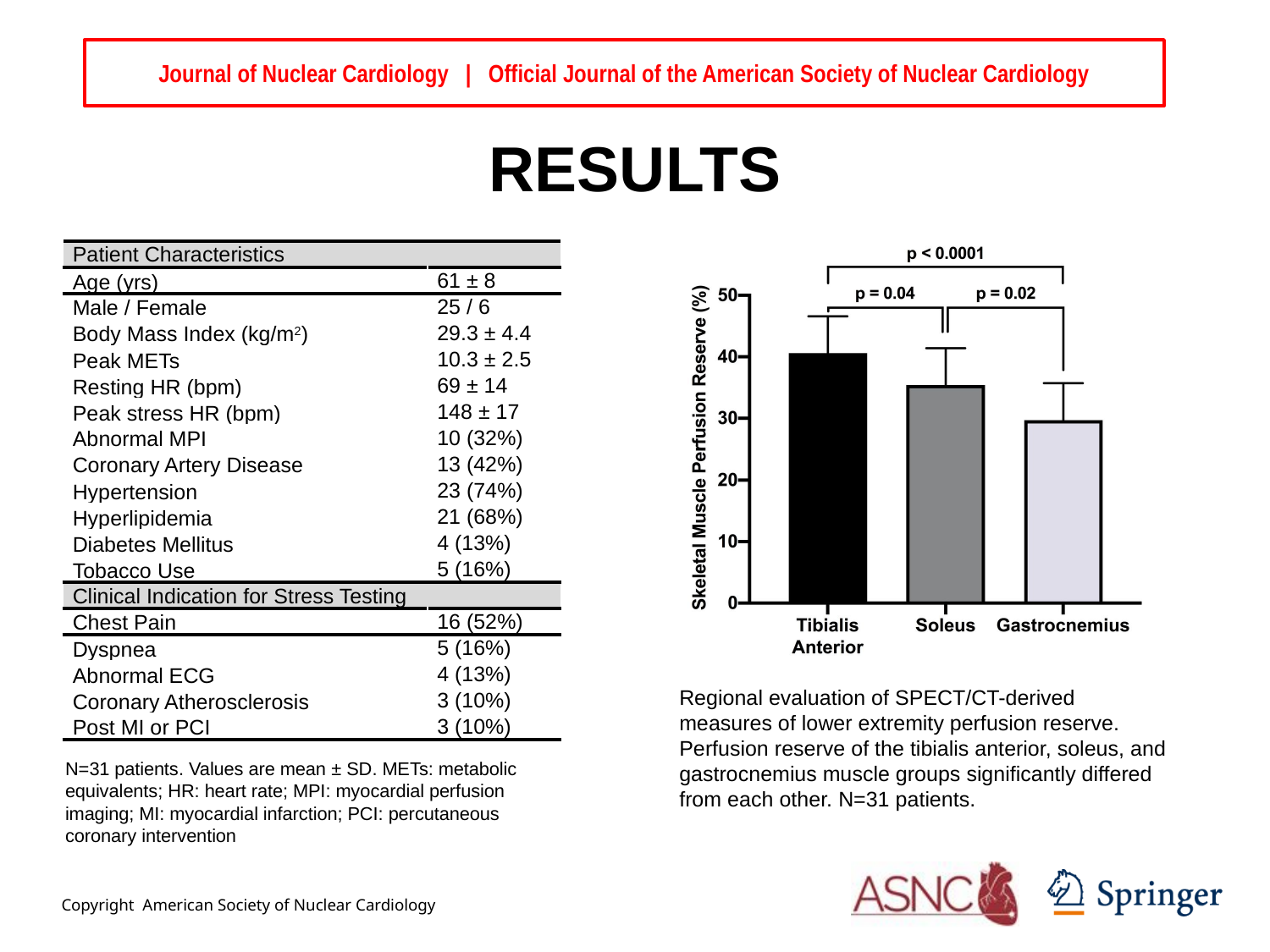

Journal of Nuclear Cardiology | Official Journal of the American Society of Nuclear Cardiology
# RESULTS
| Patient Characteristics | |
| --- | --- |
| Age (yrs) | 61 ± 8 |
| Male / Female | 25 / 6 |
| Body Mass Index (kg/m2) | 29.3 ± 4.4 |
| Peak METs | 10.3 ± 2.5 |
| Resting HR (bpm) | 69 ± 14 |
| Peak stress HR (bpm) | 148 ± 17 |
| Abnormal MPI | 10 (32%) |
| Coronary Artery Disease | 13 (42%) |
| Hypertension | 23 (74%) |
| Hyperlipidemia | 21 (68%) |
| Diabetes Mellitus | 4 (13%) |
| Tobacco Use | 5 (16%) |
| Clinical Indication for Stress Testing | |
| Chest Pain | 16 (52%) |
| Dyspnea | 5 (16%) |
| Abnormal ECG | 4 (13%) |
| Coronary Atherosclerosis | 3 (10%) |
| Post MI or PCI | 3 (10%) |
Regional evaluation of SPECT/CT-derived measures of lower extremity perfusion reserve. Perfusion reserve of the tibialis anterior, soleus, and gastrocnemius muscle groups significantly differed from each other. N=31 patients.
N=31 patients. Values are mean ± SD. METs: metabolic equivalents; HR: heart rate; MPI: myocardial perfusion imaging; MI: myocardial infarction; PCI: percutaneous coronary intervention
Copyright American Society of Nuclear Cardiology

## Slide 5
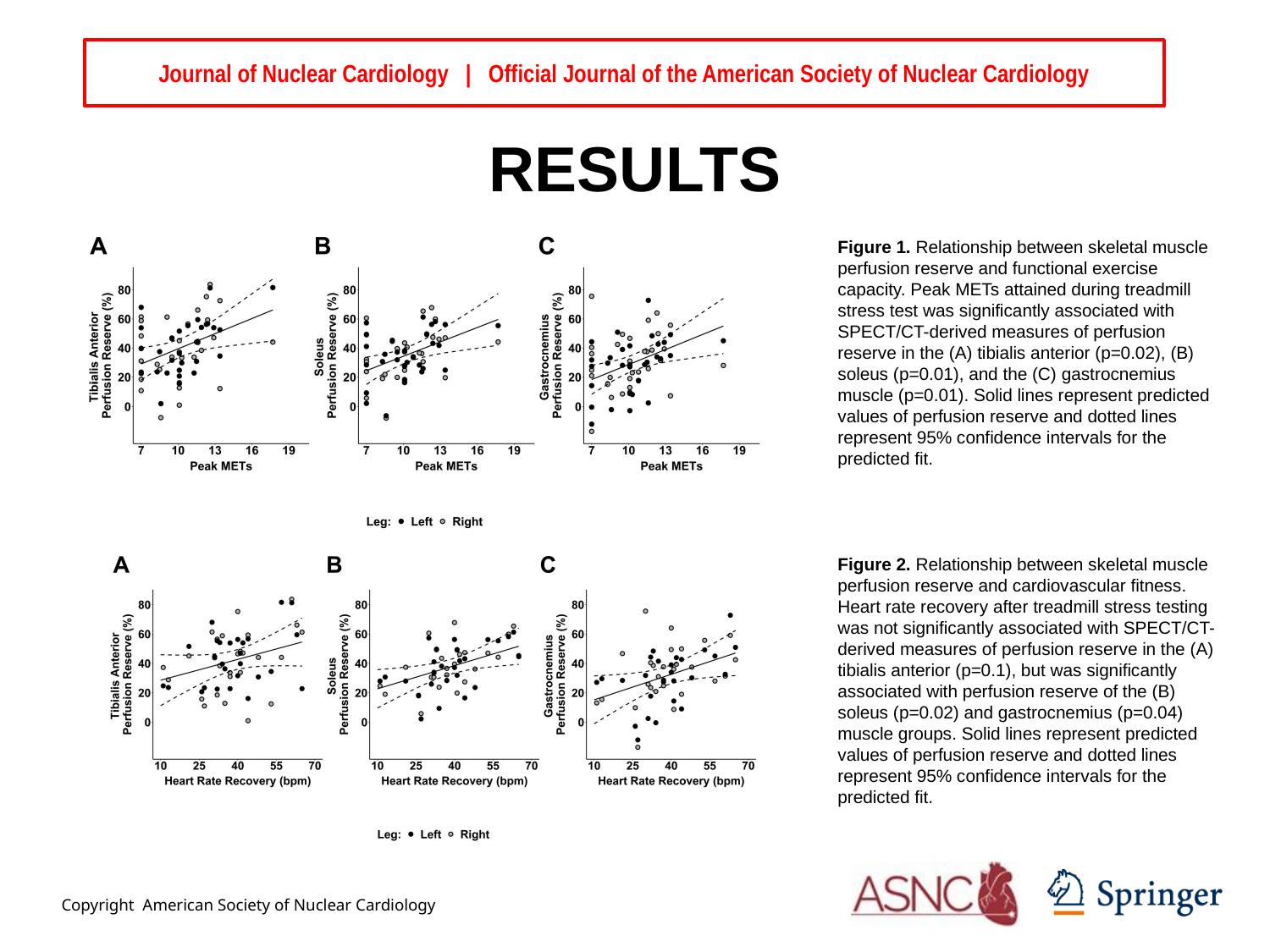

Journal of Nuclear Cardiology | Official Journal of the American Society of Nuclear Cardiology
# RESULTS
Figure 1. Relationship between skeletal muscle perfusion reserve and functional exercise capacity. Peak METs attained during treadmill stress test was significantly associated with SPECT/CT-derived measures of perfusion reserve in the (A) tibialis anterior (p=0.02), (B) soleus (p=0.01), and the (C) gastrocnemius muscle (p=0.01). Solid lines represent predicted values of perfusion reserve and dotted lines represent 95% confidence intervals for the predicted fit.
Figure 2. Relationship between skeletal muscle perfusion reserve and cardiovascular fitness. Heart rate recovery after treadmill stress testing was not significantly associated with SPECT/CT-derived measures of perfusion reserve in the (A) tibialis anterior (p=0.1), but was significantly associated with perfusion reserve of the (B) soleus (p=0.02) and gastrocnemius (p=0.04) muscle groups. Solid lines represent predicted values of perfusion reserve and dotted lines represent 95% confidence intervals for the predicted fit.
Copyright American Society of Nuclear Cardiology

## Slide 6
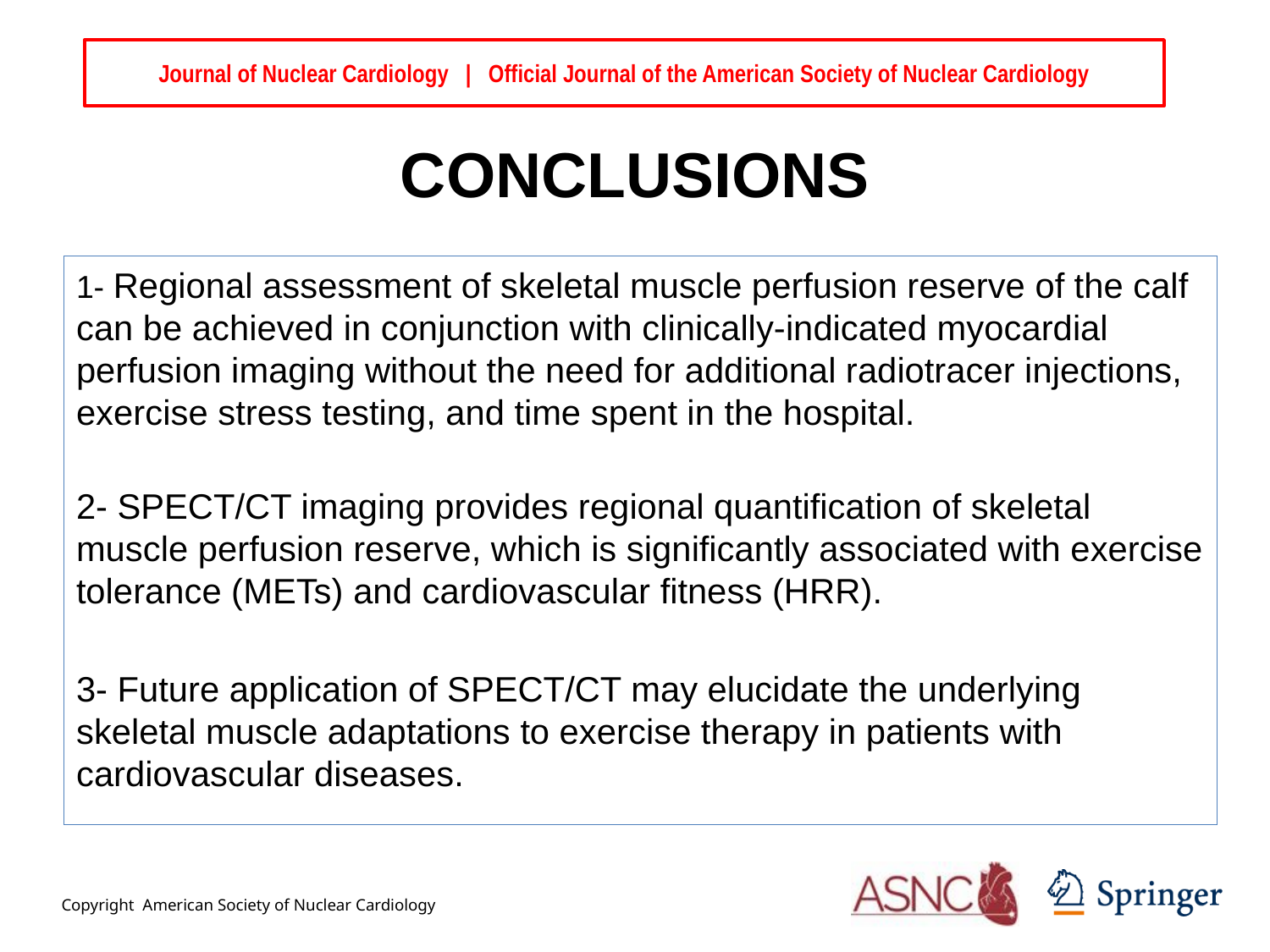

Journal of Nuclear Cardiology | Official Journal of the American Society of Nuclear Cardiology
# CONCLUSIONS
1- Regional assessment of skeletal muscle perfusion reserve of the calf can be achieved in conjunction with clinically-indicated myocardial perfusion imaging without the need for additional radiotracer injections, exercise stress testing, and time spent in the hospital.
2- SPECT/CT imaging provides regional quantification of skeletal muscle perfusion reserve, which is significantly associated with exercise tolerance (METs) and cardiovascular fitness (HRR).
3- Future application of SPECT/CT may elucidate the underlying skeletal muscle adaptations to exercise therapy in patients with cardiovascular diseases.
Copyright American Society of Nuclear Cardiology
