# Supplementary material for: SPECT/CT imaging of lower extremity perfusion reserve: A non-invasive correlate to exercise tolerance and cardiovascular fitness in patients undergoing clinically indicated myocardial perfusion imaging
Source: J Nucl Cardiol. 2020 Jan 14;27(6):1923–33. doi: 10.1007/s12350-019-02019-w (PMC7749094; doi:10.1007/s12350-019-02019-w)
Supplement: Supplementary file 3 — Electronic supplementary material 3 (DOCX 12 kb) [file 12350_2019_2019_MOESM3_ESM.docx]

Skeletal muscle perfusion reserve is significantly associated with exercise tolerance and cardiovascular fitness in patients undergoing clinically-indicated stress testing.
