# Supplementary figures and images for: SPECT/CT imaging of lower extremity perfusion reserve: A non-invasive correlate to exercise tolerance and cardiovascular fitness in patients undergoing clinically indicated myocardial perfusion imaging
Source: J Nucl Cardiol. 2020 Jan 14;27(6):1923–33. doi: 10.1007/s12350-019-02019-w (PMC7749094; doi:10.1007/s12350-019-02019-w)

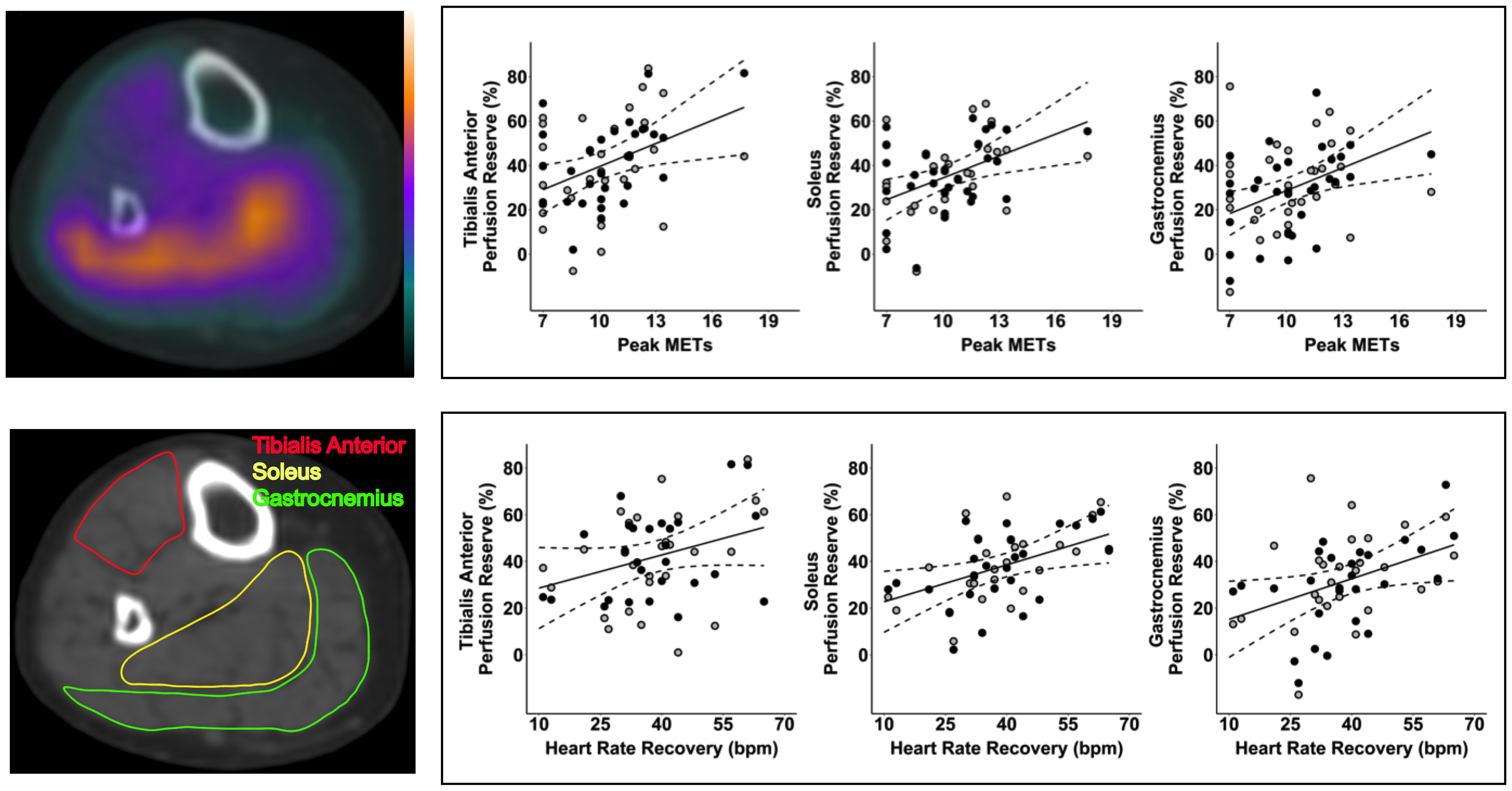

Supplement: Supplementary file 4 — Electronic supplementary material 4 (TIFF 2992 kb) [file 12350_2019_2019_MOESM4_ESM.tiff]
